# Association between SARS-CoV-2 vaccination and healthcare contacts for menstrual disturbance and bleeding in women before and after menopause: nationwide, register based cohort study 

**DOI:** 10.1136/bmj-2023-074778

**Published:** 2023-05-03

**Authors:** Rickard Ljung, YiYi Xu, Anders Sundström, Susannah Leach, Ebba Hallberg, Maria Bygdell, Maria Larsson, Veronica Arthurson, Magnus Gisslén, Rolf Gedeborg, Fredrik Nyberg

**Affiliations:** 1 Division of Use and Information, Swedish Medical Products Agency, Uppsala, Sweden; 2 Institute of Environmental Medicine, Karolinska Institutet, Stockholm, Sweden; 3 School of Public Health and Community Medicine, Institute of Medicine, Sahlgrenska Academy, University of Gothenburg, Gothenburg, Sweden; 4 Department of Microbiology and Immunology, Institute of Biomedicine, Sahlgrenska Academy, University of Gothenburg, Gothenburg, Sweden; 5 Department of Clinical Pharmacology, Sahlgrenska University Hospital, Gothenburg, Sweden; 6 Department of Internal Medicine and Clinical Nutrition, Institute of Medicine, The Sahlgrenska Academy, University of Gothenburg, Gothenburg, Sweden; 7 Department of Infectious Diseases, Institute of Biomedicine, Sahlgrenska Academy, University of Gothenburg, Gothenburg, Sweden; 8 Region Västra Götaland, Department of Infectious Diseases, Sahlgrenska University Hospital, Gothenburg, Sweden; 9 Division of Licensing, Swedish Medical Products Agency, PO Box 26, 751 03 Uppsala, Sweden; 10 Department of Surgical Sciences, Uppsala University, Uppsala, Sweden

## Abstract

**Objectives:**

To evaluate the risks of any menstrual disturbance and bleeding following SARS-CoV-2 vaccination in women who are premenopausal or postmenopausal.

**Design:**

A nationwide, register based cohort study.

**Setting:**

All inpatient and specialised outpatient care in Sweden from 27 December 2020 to 28 February 2022. A subset covering primary care for 40% of the Swedish female population was also included.

**Participants:**

2 946 448 Swedish women aged 12-74 years were included. Pregnant women, women living in nursing homes, and women with history of any menstruation or bleeding disorders, breast cancer, cancer of female genital organs, or who underwent a hysterectomy between 1 January 2015 and 26 December 2020 were excluded.

**Interventions:**

SARS-CoV-2 vaccination, by vaccine product (BNT162b2, mRNA-1273, or ChAdOx1 nCoV-19 (AZD1222)) and dose (unvaccinated and first, second, and third dose) over two time windows (one to seven days, considered the control period, and 8-90 days).

**Main outcome measures:**

Healthcare contact (admission to hospital or visit) for menstrual disturbance or bleeding before or after menopause (diagnosed with the International Statistical Classification of Diseases and Related Health Problems, Tenth Revision codes N91, N92, N93, N95).

**Results:**

2 580 007 (87.6%) of 2 946 448 women received at least one SARS-CoV-2 vaccination and 1 652 472 (64.0%) 2 580 007 of vaccinated women received three doses before the end of follow-up. The highest risks for bleeding in women who were postmenopausal were observed after the third dose, in the one to seven days risk window (hazard ratio 1.28 (95% confidence interval 1.01 to 1.62)) and in the 8-90 days risk window (1.25 (1.04 to 1.50)). The impact of adjustment for covariates was modest. Risk of postmenopausal bleeding suggested a 23-33% increased risk after 8-90 days with BNT162b2 and mRNA-1273 after the third dose, but the association with ChAdOx1 nCoV-19 was less clear. For menstrual disturbance or bleeding in women who were premenopausal, adjustment for covariates almost completely removed the weak associations noted in the crude analyses.

**Conclusions:**

Weak and inconsistent associations were observed between SARS-CoV-2 vaccination and healthcare contacts for bleeding in women who are postmenopausal, and even less evidence was recorded of an association for menstrual disturbance or bleeding in women who were premenopausal. These findings do not provide substantial support for a causal association between SARS-CoV-2 vaccination and healthcare contacts related to menstrual or bleeding disorders.

## Introduction

Menstrual disturbances such as excessive, frequent, and irregular menstruation or absent, scant, and rare menstruation have been reported in association with SARS-CoV-2 vaccines. The US Vaccine Adverse Event Reporting System, the UK Medicines and Healthcare Products Regulatory Agency’s Yellow Card surveillance scheme, and the Swedish Medical Products Agency have received many reports of menstrual disturbance after SARS-CoV-2 vaccination via their respective pharmacovigilance systems.^1^ ^2^ ^3^

Several studies on self-reported menstruation cycles after SARS-CoV-2 vaccination, from survey data and a menstrual cycle tracking app, indicate changes in menstruation cycles.^4^ ^5^ ^6^ ^7^ ^8^ ^9^ A link between SARS-CoV-2 vaccination and menstrual disturbance has also been widely discussed on social media.^10^ However, menstrual cycles vary naturally and minor menstrual disturbances are generally not considered to be of clinical importance. Changes can, however, generate considerable distress in the affected women, especially during a mass vaccination campaign when concerns are raised about adverse reactions that might not yet be well characterised.^11^ The Pharmacovigilance Risk Assessment Committee of the European Medicines Agency has recommended listing heavy menstrual bleeding as a side effect of unknown frequency in the product information for the SARS-CoV-2 mRNA vaccines. The recommendation follows a review of the available evidence, including cases reported during clinical trials, cases spontaneously reported in Eudravigilance, and findings from the medical literature.^12^ Previously, investigations researched concerns about menstrual disturbances from other vaccines (eg, against human papillomavirus), but no such association was established.^13^ ^14^ ^15^

Pharmacovigilance systems relying on self-reporting are useful for identifying potential safety signals but not suited for quantifying the frequency of health event occurrence or estimating the strength of the potential association. To characterise and quantify suspected adverse effects of SARS-CoV-2 vaccines, outside what is detected in clinical trials, individual level data from large observational studies are needed.^16^

In a nationwide cohort study in Sweden, we evaluated the risks of menstrual disturbance and bleeding after SARS-CoV-2 vaccination in women who were before or after menopause. High quality data from nationwide registers enabled us to evaluate the risk by vaccine product and vaccination dose number.

## Material and methods

### Data sources

For all individuals, we linked data from Swedish national and regional registers as an analysis within the RECOVAC (register-based large-scale national population study to monitor SARS-CoV-2 vaccination effectiveness and safety) study, which is within the larger project of SCIFI-PEARL (Swedish Covid-19 Investigation for Future Insights—a Population Epidemiology Approach using Register Linkage), described in detail elsewhere.^17^ A complete medical history from 1 January 2015 was obtained from the national patient register and drug history for prescription drugs from 1 January2018 from the national prescribed drug register.^18^ ^19^ History of cancer was obtained from the national cancer register.^20^ Sociodemographic data including education, family situation, income, and occupation data from 2015 were obtained from Statistics Sweden.^21^ Information about pregnancy was obtained from the national medical birth register. Information about older patients living at special care facilities or receiving home care services was obtained from the register of social service interventions for the elderly and the disabled.^22^

Vaccination data, including vaccine product, dose number, and date of vaccination, were obtained from the national vaccination register.^23^ Positive results from SARS-CoV-2 polymerase chain reaction tests were identified from SmiNet, the national register of notifiable communicable diseases.^24^ We obtained diagnoses of menstrual disturbance and bleeding in women before or after menopause from healthcare contacts registered as outpatient specialist visits or inpatient stays from the national patient register. The risk of having any diagnosis of menstrual disturbance, bleeding before and after menopause after contact with a healthcare service is hereafter referred to as risk of menstruation disorders. In Sweden, women with gynaecological issues will often, especially in urban areas, turn directly to gynaecological specialist care. However, in a subpopulation, we were also able to include information on primary care visits. Thus, for women living in the two largest metropolitan areas (Stockholm region and Västra Götaland region), diagnoses were additionally obtained from regional primary healthcare registers. The date and cause of death were obtained from the register of the total population and the national cause of death register.^25^ ^26^

### Study population

The study included all women aged 12-74 years who were residing in Sweden on 1 January 2018 (to ensure previous comorbidities are accounted for), and still resident in the country on 27 December 2020, when the SARS-CoV-2 vaccine campaign started in Sweden. Data for sex was taken from information in the registry rather than from patient reported gender. The exclusion criteria were women living at special care facilities (5927 women (0.15% of those aged 12-74)) until 31 December 2020, and individuals who were pregnant or had a history of any menstruation disorders, breast cancer, cancer of the female genital organs, or who underwent a hysterectomy between 1 January 2015 (the maximum period of stored history from the register data) and 26 December 2020.

### Study period, exposures, and risk windows

The study period was from 27 December 2020 to 28 February 2022. Exposure variables were each dose of any vaccine, and several different risk periods were applied. In the main analyses, we used two mutually exclusive risk periods, one to seven days and 8-90 days after vaccination. The first seven days were deemed to be a negative control period. The time needed for an unknown pathological mechanism to manifest need to be considered, the symptoms then develop to become sufficiently worrying for the woman to seek medical attention, and the healthcare system them provides an appointment or admission, which results in a diagnosis. For menstrual disturbances, a woman is unlikely to notice any effects and be able to get an acute appointment within the first week. As menstrual cycles are around 28 days, we anticipated that a women would be delayed in deciding to seek medical attention for any disturbances. Hence, the 90 day window allows for two cycles and an additional month for the individual to get an appointment, including a potential additional interval in getting an appointment with a gynaecologist. We assessed the risk of menstruation disorders in each risk period after the administration date of the first, second, and third dose with any vaccine. Stratified analyses were performed for three specific vaccine brands used in Sweden, BNT162b2 (Pfizer-BioNTech), mRNA-1273 (Moderna), and ChAdOx1 nCoV-19 (AZD1222) (AstraZeneca). In sensitivity analyses, we also estimated risk with follow-ups at days seven, 28, and 90 starting the day after exposure date. We performed our main analyses for the whole study population and additional analyses in a subpopulation living in the two largest metropolitan areas (Stockholm region and Västra Götaland region), where regional primary healthcare data were also available.

To contextualise the results, we also estimated the risk for menstruation disorders after a SARS-CoV-2 infection in women who were not vaccinated. The study period for this analysis was from 1 August 2020 (when full-scale testing was implemented in Sweden) to 26 December 2020 (when vaccinations started). In this analysis, we included all female individuals aged 12-74 years who were residing in Sweden on 1 January 2018 and 1 August 2020, who were not pregnant, did not live in nursing home on 1 August 2020, and did not have the previously mentioned comorbidities within five years before August 2020. We also studied the risk of menstruation disorders during follow-up at days seven, 28, and 90 after the first positive test result of SARS-CoV-2 infection.

### Outcomes

We studied three different menstruation disorders in different restricted age ranges (defined to include premenopausal or postmenopausal women when relevant for the respective outcomes). We identified only incident cases by using the first recording of a primary diagnosis, according to the Swedish clinical modification of the 10th revision of the International Statistical Classification of Diseases and Related Health Problems (ICD-10-SE), in one of the registers to define the outcome. Hence, outcomes were based on a healthcare contact (admission to hospital or visit) where a physician registered any of the diagnoses under study. All healthcare contacts with any of the diagnoses under study were included in the analyses including primary care, regardless of whether these contacts were related to a physician or other healthcare worker visit. The date of diagnosis was regarded as a proxy for date of onset because we have no means to assess the true start of symptoms. We studied postmenopausal bleeding in women of 45-74 years, using ICD-10-SE code N95.0.

We also studied menstrual disturbance in women aged 12-49 years, using ICD-10-SE codes N91 and N92.

Additionally, we studied premenopausal bleeding in women aged 12-49 years, using ICD-10-SE codes N93.8 and N93.9. For the Stockholm region and Västra Götaland region, the ICD-10-SE-P (for primary care) code N93 was additionally used.

### Covariates

Covariates included in the full models were age (cubic spline with four knots), country of birth (Sweden/other countries), employed as a healthcare worker (yes/no), marital status (married/not married), education (primary, secondary, tertiary, undetermined), number of primary care visits, number of specialist outpatient visits, and days of inpatient stay, during 2018-19, as well as prior comorbidities and treatments (each yes/no; listed in supplement table S1, directed acyclic graphs, supplement DAG S1, and supplement DAG S2) (table 1, supplement table S2).

**Table 1:** Distribution of characteristics related to demographics and medical history, by vaccine status. All women were unvaccinated at the baseline, and they can contribute with person-time to more than one vaccine status group

Covariates	Baseline (all unvaccinated) (n=2 946 448)	At least one dose (n=2 580 007)	At least two doses (n=2 515 868)	Three doses and more (n=1 652 472)
Age, median (IQR)	44 (24-58)	46 (25-59)	46 (26-59)	53 (41-63)
Employed as a healthcare worker:				
No	2 018 128 (68.5)	1 740 484 (67.5)	1 690 984 (67.2)	1 057 348 (64.0)
Yes	928 320 (31.5)	839 523 (32.5)	824 884 (32.8)	595 124 (36.0)
Country of birth:				
Sweden	2 383 529 (80.9)	2 153 373 (83.5)	2 107 976 (83.8)	1 432 112 (86.7)
Outside Sweden	562 919 (19.1)	426 634 (16.5)	407 892 (16.2)	220 360 (13.3)
Education:				
Primary	416 303 (14.1)	354 647 (13.7)	342 635 (13.6)	204 655 (12.4)
Secondary	1 076 764 (36.5)	957 364 (37.1)	940 376 (37.4)	689 049 (41.7)
Tertiary	1 077 071 (36.6)	989 890 (38.4)	978 262 (38.9)	743 930 (45)
Unknown	376 310 (12.8)	278 106 (10.8)	254 595 (10.1)	14 838 (0.9)
Cardiovascular disease:				
No	2 850 349 (96.7)	2 491 633 (96.6)	2 428 912 (96.5)	1 580 841 (95.7)
Yes	96 099 (3.3)	88 374 (3.4)	86 956 (3.5)	71 631 (4.3)
Stroke or transient ischaemic attack:				
No	2 927 167 (99.3)	2 562 162 (99.3)	2 498 227 (99.3)	1 637 146 (99.1)
Yes	19 281 (0.7)	17 845 (0.7)	17 641 (0.7)	15 326 (0.9)
Diabetes (type 1 and 2):				
No	2 835 636 (96.2)	2 477 521 (96.0)	2 414 737 (96.0)	1 568 459 (94.9)
Yes	110 812 (3.8)	102 486 (4.0)	101 131 (4.0)	84 013 (5.1)
Chronic pulmonary disease:				
No	2 928 408 (99.4)	2 563 464 (99.4)	2 499 620 (99.4)	1 638 670 (99.2)
Yes	18 040 (0.6)	16 543 (0.6)	16 248 (0.6)	13 802 (0.8)
Asthma:				
No	2 877 204 (97.6)	2 518 549 (97.6)	2 456 384 (97.6)	1 615 853 (97.8)
Yes	69 244 (2.4)	61 458 (2.4)	59 484 (2.4)	36 619 (2.2)
Chronic kidney disease:				
No	2 908 552 (98.7)	2 546 491 (98.7)	2 483 198 (98.7)	1 629 523 (98.6)
Yes	37 896 (1.3)	33 516 (1.3)	32 670 (1.3)	22 949 (1.4)
Cancer:				
No	2 886 227 (98.0)	2 523 106 (97.8)	2 459 511 (97.8)	1 602 926 (97.0)
Yes	60 221 (2.0)	56 901 (2.2)	56 357 (2.2)	49 546 (3.0)
Coagulation disorders:				
No	1 139 856 (99.2)	1 038 321 (99.2)	1 018 983 (99.2)	702 468 (99.2)
Yes	8821 (0.8)	7890 (0.8)	7717 (0.8)	5634 (0.8)
Polycystic ovary syndrome:				
No	1 139 698 (99.2)	1 038 724 (99.3)	1 019 459 (99.3)	704 123 (99.4)
Yes	8979 (0.8)	7487 (0.7)	7241 (0.7)	3979 (0.6)
Thyroid diseases:				
No	2 728 628 (92.6)	2 380 747 (92.3)	2 319 269 (92.2)	1 494 707 (90.5)
Yes	217 820 (7.4)	199 260 (7.7)	196 599 (7.8)	157 765 (9.5)
Pituitary disorders:				
No	1 142 025 (99.4)	1 040 378 (99.4)	1 020 982 (99.4)	704 115 (99.4)
Yes	6652 (0.6)	5833 (0.6)	5718 (0.6)	3987 (0.6)
Uterine polyps or fibroids:				
No	1 119 516 (97.5)	1 020 167 (97.5)	1 001 063 (97.5)	688 302 (97.2)
Yes	29 161 (2.5)	26 044 (2.5)	25 637 (2.5)	19 800 (2.8)
Endometriosis:				
No	913 984 (79.6)	837 134 (80.0)	821 956 (80.1)	566 195 (80.0)
Yes	234 693 (20.4)	209 077 (20.0)	204 744 (19.9)	141 907 (20.0)
Pelvic inflammatory diseases:				
No	1 052 420 (91.6)	963 202 (92.1)	946 222 (92.2)	658 303 (93.0)
Yes	96 257 (8.4)	83 009 (7.9)	80 478 (7.8)	49 799 (7.0)
Obesity:				
No	2 898 410 (98.4)	2 537 952 (98.4)	2 475 086 (98.4)	1 624 829 (98.3)
Yes	48 038 (1.6)	42 055 (1.6)	40 782 (1.6)	27 643 (1.7)
Autoimmune diseases:				
No	2 883 402 (97.9)	2 522 085 (97.8)	2 458 815 (97.7)	1 606 176 (97.2)
Yes	63 046 (2.1)	57 922 (2.2)	57 053 (2.3)	46 296 (2.8)
Menopausal hormone:				
No	910 720 (79.3)	820 835 (78.5)	803 415 (78.3)	518 113 (73.2)
Yes	237 957 (20.7)	225 376 (21.5)	223 285 (21.7)	189 989 (26.8)
Contraception:				
No	673 527 (58.6)	611 978 (58.5)	601 640 (58.6)	465 248 (65.7)
Yes	475 150 (41.4)	434 233 (41.5)	425 060 (41.4)	242 854 (34.3)
Anticoagulants:				
No	2 813 692 (95.5)	2 455 434 (95.2)	2 392 597 (95.1)	1 544 641 (93.5)
Yes	132 756 (4.5)	124 573 (4.8)	123 271 (4.9)	107 831 (6.5)
Antidepressant treatment:				
No	2 556 629 (86.8)	2 222 691 (86.2)	2 165 607 (86.1)	1 397 733 (84.6)
Yes	389 819 (13.2)	357 316 (13.8)	350 261 (13.9)	254 739 (15.4)
Tranexamic acid:				
No	1 137 176 (99.0)	1 035 938 (99.0)	1 016 703 (99.0)	701 847 (99.1)
Yes	11 501 (1.0)	10 273 (1.0)	9997 (1.0)	6255 (0.9)
Oral corticosteroids:				
No	2 810 256 (95.4)	2 455 195 (95.2)	2 393 249 (95.1)	1 556 863 (94.2)
Yes	136 192 (4.6)	124 812 (4.8)	122 619 (4.9)	95 609 (5.8)
Epilepsy medication:				
No	1 063 528 (92.6)	969 199 (92.6)	951 321 (92.7)	651 465 (92.0)
Yes	85 149 (7.4)	77 012 (7.4)	75 379 (7.3)	56 637 (8.0)
NSAIDs:				
No	801 144 (69.7)	729 467 (69.7)	715 586 (69.7)	475 232 (67.1)
Yes	347 533 (30.3)	316 744 (30.3)	311 114 (30.3)	232 870 (32.9)
Numbers of specialist outpatient visits, median (IQR)	1 (0-3)	1 (0-3)	1 (0-3)	1 (0-3)
Days of inpatient stay, median (IQR)	0 (0-0)	0 (0-0)	0 (0-0)	0 (0-0)
Number of primary care visits, median (IQR)	4 (1-12)	4 (1-12)	4 (1-13)	5 (1-14)

Data are number (percentage), unless otherwise stated. IQR=interquartile range; NSAIDs=Non-steroidal anti-inflammatory drugs.

### Statistical analysis

Cox proportional hazards models with time varying exposure were used, where each woman’s follow-up time was divided according to her vaccination status (unvaccinated, first dose, second dose, and third dose), and then at each risk window (one to seven days and 8-90 days after each dose in the main analyses, and within days seven, 28, or 90 in the sensitivity analyses). Each individual was followed up from 27 December 2020 until the earliest of the outcome of interest, end of each risk window, or a censoring event (defined as receiving a second, third, or fourth dose of any vaccine, emigration, death, or end of study on 28 February 2022). An individual contributed person-time as unvaccinated until the first vaccination. After each vaccination dose, individuals contributed person-time in each corresponding risk window of interest (ie, exposed risk time). We also restricted analyses to the subpopulation where primary care data were available. Additionally, we performed sensitivity analyses limited to women without previous hormone treatment, and in women without a prior diagnosis of coagulation disease or a filled prescription for anticoagulants.

In the complementary analyses, to assess the risk for menstruation disorders at days seven, 28, or 90 after a covid-19 infection in unvaccinated women, each woman’s follow-up time was divided according to covid-19 infection status (no infection period and period after first positive SARS-CoV-2 test) and then at each risk window (within days 7, 28, or 90 after first positive test). Each woman was followed up from 1 August 2020 until the earliest of the outcome of interest, end of each risk window, or a censoring event (ie, emigration, death, or end of study on 26 December 2020).

Hazard ratios with 95% confidence intervals were estimated from Cox models. We report results from a crude model without any adjustment for covariates, and a full model adjusted for all covariates listed previously.

### Patient and public involvement

Patients were not directly involved in the study. However, the rationale for the study was around 8000 (November 2022) reports of suspected adverse drug reactions regarding menstrual disturbances that were reported to the Swedish Medical Products Agency. Approximately 90% of the suspected adverse drug reactions were reported by consumers.

## Results

### Descriptive analyses 

**Fig 1:**
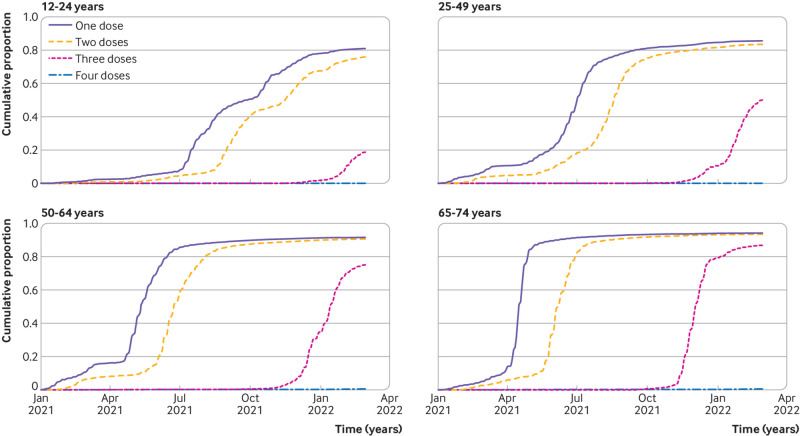
Cumulative proportion of vaccine uptake (up to four doses) in different age groups, between 1 January 2020 and 28 February 2022, among women in a Swedish population cohort. Vaccination started on 27 December 2020 for the oldest age group and patients at highest risk

In total, 2 946 448 girls and women aged 12-74 years were included in the vaccination analyses. Of these, 2 580 007 (87.6%) received at least one SARS-CoV-2 vaccination before the end of follow-up on 28 February 2022. Among the vaccinated, 1 652 472 (64.0%) of 2 580 007 women received three doses, but this proportion varied by age (fig 1). Participants’ demographics and medical history are presented in table 1 and table S2 by vaccine status. Women can contribute with person-time to more than one vaccine status group.

More than 99% of menstrual disturbance (19 329/19 443 cases in the National Patient Register) or bleeding disorder diagnoses (9370/9407 cases) in the overall study population were from specialist outpatient care. In the subpopulation where primary care data were available (n=1 156 260, approximately 40% of the Swedish female population), about 11% (666/6207 cases) of the diagnoses reflecting premenopausal and postmenopausal bleeding, and 19% (2119/11 344 cases) of diagnoses of menstrual disturbance were recorded in primary healthcare. Crude annual rates of the outcomes during the study period of 2015-22 were of similar magnitude (supplement table S3).

For the analyses of menstruation or bleeding disorders after a positive SARS-CoV-2 test, 754 991 (25.7%) of 2 942 544 women tested positive for SARS-CoV-2 during the study period of 1 August 2020 to 26 December 2020.

### Menstrual disturbance and bleeding disorders after vaccination

#### Postmenopausal bleeding

Adjusted hazard ratio comparing the risk for postmenopausal bleeding after vaccination with any dose compared with unvaccinated periods was 1.12 (95% confidence interval 1.00 to 1.25) in the one to seven days risk window and 1.14 (1.06 to 1.23) in the 8-90 days risk window (table 2, supplement figure S1). The impact of adjustment for covariates was modest. The highest risks were observed after the third dose, both in the one to seven days risk window (1.28 (1.01 to 1.62)) and the 8-90 days risk window (1.25 (1.04 to 1.50)). The precision of these estimates was overall good. The results from the subpopulation with primary care data showed similar pattern to the main analyses, but with generally lower risk estimates (table 3, supplement figure S2). After restriction to women without prior hormone treatment, increased risks were observed after the third dose in both risk windows, with slightly higher estimates than in the main analyses (table 2, supplement table S4). The strongest association was reported in the third dose in the one to seven days risk window (1.48 (1.12 to 1.94)); similar risks were also observed in the subpopulation with primary care data, most obviously for the second dose in the 8-90 days risk window (supplement table S4). Exclusion of women with prior coagulation disorders did not change the results notably compared with the main analyses (table 2, supplement table S5).

**Table 2:** Hazard ratios (HR) with 95% confidence interval (CI) for menstruation disorders after each dose in one to seven days and 8-90 days risk windows, among women in a Swedish population cohort

Risk windows	Person-years	Cases	Incidence rate (per 100 000 person-years)	Crude model,* HR (95% CI)	Full model,† HR (95% CI)
**Postmenopausal bleeding (45-74 years, n=1** **561** **429)**					
Unvaccinated	646 133	3144	486.6	ref	ref
Any dose:					
1-7 days	77 501	416	536.8	1.19 (1.06 to 1.33)	1.12 (1.00 to 1.25)
8-90 days	665 572	3401	511.0	1.21 (1.13 to 1.29)	1.14 (1.06 to 1.23)
Dose 1:					
1-7 days	27 379	166	606.3	1.20 (1.02 to 1.41)	1.15 (0.98 to 1.35)
8-90 days	159 069	844	530.6	1.14 (1.04 to 1.25)	1.08 (0.98 to 1.19)
Dose 2:					
1-7 days	27 216	122	448.3	1.06 (0.88 to 1.29)	0.98 (0.81 to 1.19)
8-90 days	320 329	1561	487.3	1.22 (1.11 to 1.34)	1.14 (1.03 to 1.25)
Dose 3:					
1-7 days	22 907	128	558.8	1.45 (1.14 to 1.84)	1.28 (1.01 to 1.62)
8-90 days	186 174	996	535.0	1.40 (1.17 to 1.67)	1.25 (1.04 to 1.50)
**Menstrual disturbance (12-49 years, n=1** **634** **294)**					
Unvaccinated	1 067 762	9615	900.5	ref	ref
Any dose:					
1-7 days	62 278	674	1082.2	1.41 (1.29 to 1.52)	1.13 (1.04 to 1.23)
8-90 days	480 493	4970	1034.4	1.38 (1.32 to 1.44)	1.06 (1.01 to 1.11)
Dose 1:					
1-7 days	26 034	288	1106.2	1.49 (1.32 to 1.68)	1.26 (1.11 to 1.42)
8-90 days	147 296	1364	926.0	1.29 (1.21 to 1.37)	1.07 (1.00 to 1.14)
Dose 2:					
1-7 days	24 969	250	1001.2	1.21 (1.06 to 1.37)	1.04 (0.91 to 1.18)
8-90 days	281 999	2981	1057.1	1.33 (1.26 to 1.40)	1.04 (0.98 to 1.10)
Dose 3:					
1-7 days	11 274	136	1206.3	1.34 (1.11 to 1.62)	1.02 (0.84 to 1.23)
8-90 days	51 198	625	1220.8	1.43 (1.27 to 1.62)	1.00 (0.89 to 1.13)
**Premenopausal bleeding (12-49 years, n=1** **634** **294)**					
Unvaccinated	1 070 500	1865	174.2	ref	ref
Any dose:					
1-7 days	62 625	133	212.4	1.44 (1.2 to 1.74)	1.08 (0.90 to 1.30)
8-90 days	484 600	1002	206.8	1.43 (1.3 to 1.58)	1.01 (0.91 to 1.12)
Dose 1:					
1-7 days	26 144	54	206.6	1.40 (1.06 to 1.85)	1.14 (0.86 to 1.50)
8-90 days	148 118	273	184.3	1.32 (1.14 to 1.51)	1.01 (0.88 to 1.16)
Dose 2:					
1-7 days	25 096	46	183.3	1.22 (0.90 to 1.65)	0.96 (0.71 to 1.30)
8-90 days	284 736	608	213.5	1.45 (1.29 to 1.63)	1.03 (0.92 to 1.17)
Dose 3:					
1-7 days	11 385	33	289.9	1.67 (1.13 to 2.49)	1.14 (0.77 to 1.70)
8-90 days	51 745	121	233.8	1.32 (1.00 to 1.75)	0.83 (0.63 to 1.10)

* Crude model included no covariates.

† Full model included age, country of birth, employed as a healthcare worker, marital status, education, and health seeking behaviours during 2018-19 (ie, no. of primary care visits, number of specialist outpatient visits, and days of inpatient stay), and prior comorbidities and treatments listed in supplement table S1.

**Table 3:** Hazard ratios (HR) with 95% confidence interval (CI) for menstrual disturbance and bleeding after each dose in one to seven days and 8-90 days risk windows in the subpopulation with primary care data (Stockholm region and Västra Götaland region, approximately 40% of total population), among women in a Swedish population cohort

Risk windows	Person-years	Cases	Incidence rate (per 100 000 person-years)	Crude model,* HR (95% CI)	Full model,† HR (95% CI)
**Postmenopausal bleeding (45-74 years, n=590 271)**					
Unvaccinated	252 977	1345	531.7	ref	ref
Any dose:					
1-7 days	28 623	175	611.4	1.26 (1.06 to 1.49)	1.16 (0.97 to 1.37)
8-90 days	243 943	1287	527.6	1.15 (1.03 to 1.28)	1.05 (0.94 to 1.17)
Dose 1:					
1-7 days	10 281	75	729.5	1.30 (1.02 to 1.66)	1.23 (0.96 to 1.57)
8-90 days	60 233	311	516.3	1.04 (0.89 to 1.21)	0.95 (0.81 to 1.11)
Dose 2:					
1-7 days	10 134	50	493.4	1.15 (0.85 to 1.56)	1.02 (0.75 to 1.39)
8-90 days	119 097	597	501.3	1.21 (1.04 to 1.40)	1.08 (0.93 to 1.25)
Dose 3:					
1-7 days	8209	50	609.1	1.41 (0.98 to 2.02)	1.18 (0.82 to 1.69)
8-90 days	64 613	379	586.6	1.34 (1.03 to 1.74)	1.13 (0.86 to 1.49)
**Menstrual disturbance (12-49 years, n=664 201)**					
Unvaccinated	446 270	6092	1365.1	ref	ref
Any dose:					
1-7 days	24 260	374	1 541.6	1.28 (1.15 to 1.43)	1.11 (0.99 to 1.23)
8-90 days	188 275	2802	1 488.2	1.27 (1.20 to 1.34)	1.06 (1.00 to 1.12)
Dose 1:					
1-7 days	10 331	169	1635.9	1.41 (1.21 to 1.65)	1.25 (1.07 to 1.46)
8-90 days	61 602	813	1319.8	1.16 (1.07 to 1.26)	1.03 (0.95 to 1.12)
Dose 2:					
1-7 days	9776	144	1473.0	1.15 (0.97 to 1.37)	1.05 (0.88 to 1.24)
8-90 days	109 514	1701	1553.2	1.26 (1.18 to 1.35)	1.07 (0.99 to 1.14)
Dose 3:					
1-7 days	4153	61	1468.7	1.03 (0.78 to 1.35)	0.87 (0.66 to 1.15)
8-90 days	17 159	288	1678.4	1.28 (1.08 to 1.51)	1.00 (0.85 to 1.19)
**Premenopausal bleeding (12-49 years, n=664 201)**					
Unvaccinated	449 008	1210	269.5	ref	ref
Any dose:					
1-7 days	24 459	75	306.6	1.33 (1.04 to 1.69)	1.00 (0.78 to 1.28)
8-90 days	190 173	603	317.1	1.39 (1.23 to 1.57)	0.99 (0.87 to 1.13)
Dose 1:					
1-7 days	10 398	32	307.8	1.37 (0.96 to 1.96)	1.13 (0.79 to 1.61)
8-90 days	62 149	162	260.7	1.21 (1.01 to 1.45)	0.95 (0.79 to 1.14)
Dose 2:					
1-7 days	9852	22	223.3	0.92 (0.60 to 1.42)	0.73 (0.47 to 1.13)
8-90 days	110 609	371	335.4	1.44 (1.24 to 1.67)	1.04 (0.89 to 1.22)
Dose 3:					
1-7 days	4209	21	498.9	1.62 (0.99 to 2.66)	1.15 (0.70 to 1.88)
8-90 days	17 414	70	402.0	1.27 (0.90 to 1.80)	0.83 (0.59 to 1.17)

* Crude model included no covariates.

† Full model included age, country of birth, employed as a healthcare worker, marital status, education, and health seeking behaviours during 2018-19 (ie, no. of primary care visits, no. of specialist outpatient visits, and days of inpatient stay), and prior comorbidities and treatments listed in supplement table S1.

#### Product specific risk estimates for postmenopausal bleeding

Analyses of associations from the full model with individual vaccine products suggested an increased risk of 41% (one to seven days) and 23% (8-90 days) with BNT162b2 after the third dose, as well as 14% increased risk during the 8-90 days risk window after the second dose (table 4, supplement figure S3). No increased risk was observed after the first dose with BNT162b2. For mRNA-1273, risk increased by 33% after the third dose in the 8-90 days risk window. The risk estimates for mRNA-1273 and ChAdOx1 nCoV-19 were overall imprecise (table 4, supplement figure S3).

**Table 4:** Hazard ratios (HR) with 95% confidence interval (CI) for postmenopausal bleeding after each dose in one to seven days and 8-90 days risk windows, stratified by vaccine product, among women in a Swedish population cohort

Risk windows	Person-years	Cases	Incidence rate (per 100 000 person-years)	Crude model,* HR (95% CI)	Full model,† HR (95% CI)
**BNT162b2 (Pfizer-BioNTech)**					
Unvaccinated	646 760	3144	486.1	ref	ref
Dose 1:					
1-7 days	20 466	120	586.3	1.16 (0.96 to 1.40)	1.09 (0.90 to 1.32)
8-90 days	103 775	532	512.6	1.11 (0.99 to 1.24)	1.01 (0.90 to 1.14)
Dose 2:					
1-7 days	21 006	101	480.8	1.13 (0.92 to 1.40)	1.02 (0.83 to 1.26)
8-90 days	247 223	1240	501.6	1.24 (1.13 to 1.37)	1.14 (1.04 to 1.26)
Dose 3:					
1-7 days	15 668	95	606.3	1.59 (1.23 to 2.06)	1.41 (1.09 to 1.83)
8-90 days	138 714	724	521.9	1.36 (1.13 to 1.63)	1.23 (1.02 to 1.49)
**mRNA-1273 (Moderna)**					
Unvaccinated	646 760	3144	486.1	ref	ref
Dose 1:					
1-7 days	2612	18	689.2	1.44 (0.90 to 2.03)	1.33 (0.84 to 2.13)
8-90 days	13 911	73	524.8	1.24 (0.97 to 1.58)	1.13 (0.88 to 1.44)
Dose 2:					
1-7 days	2691	5	185.8	0.45 (0.19 to 1.08)	0.41 (0.17 to 0.99)
8-90 days	31 409	150	477.6	1.23 (1.02 to 1.48)	1.12 (0.92 to 1.35)
Dose 3:					
1-7 days	7238	33	455.9	1.17 (0.80 to 1.72)	1.04 (0.71 to 1.53)
8-90 days	47 539	272	572.2	1.53 (1.22 to 1.91)	1.33 (1.06 to 1.67)
**ChAdOx1 nCoV-19 (AstraZeneca)**					
Unvaccinated	646 760	3144	486.1	ref	ref
Dose 1:					
1-7 days	4429	28	632.2	1.14 (0.78 to 1.66)	1.24 (0.85 to 1.81)
8-90 days	41 414	239	577.1	1.16 (1.01 to 1.34)	1.17 (1.01 to 1.35)
Dose 2:					
1-7 days	3518	16	454.8	1.27 (0.76 to 2.11)	1.21 (0.73 to 2.02)
8-90 days	41 671	171	410.4	1.17 (0.95 to 1.43)	1.14 (0.92 to 1.40)

* Crude model included no covariates.

† Full model included age, country of birth, employed as a healthcare worker, marital status, education, and health seeking behaviours during 2018-19 (ie, no. of primary care visits, no. of specialist outpatient visits, and days of inpatient stay), and prior comorbidities and treatments listed in supplement table S1.

#### Menstrual disturbance

The adjusted hazard ratio for menstrual disturbance after vaccination with any dose compared with unvaccinated periods was 1.13 (95% confidence interval 1.04 to 1.23) in the one to seven days risk window and 1.06 (1.01 to 1.11) in the 8-90 days risk window. Adjustment for covariates strongly attenuated or almost completely removed the weak associations noted in the dose specific crude analyses (table 2, supplement figure S1). The strongest adjusted association observed was a 26% increased risk of menstrual disturbance among women aged 12-49 years in the one to seven days risk window (1.26 (1.11 to 1.42)) after the first dose. The precision of these estimates was good overall. The results from the subpopulation with primary care data were largely similar to the main analyses (table 3, supplement figure S2). Similarly, product specific risk estimates were largely consistent with the overall risk estimates (table S6, supplement figure S3).

#### Premenopausal bleeding

The adjusted hazard ratio for premenopausal bleeding after vaccination with any dose compared with unvaccinated periods was 1.08 (95% confidence interval 0.90 to 1.30) in the one to seven days risk windows and 1.01 (0.91 to 1.12) for the 8-90 days risk windows. Adjustment for covariates almost completely removed the associations reported in the crude analyses (table 2, supplement figure S1). The estimates were more imprecise compared with the other outcomes because of fewer observed events. The strongest associations observed, although not significant, were a 14% increased risk in the one to seven days risk window both after the first dose (1.14 (0.86 to 1.50)) and the third dose (1.14 (0.77 to 1.70)). No increased risk was observed after the second dose (0.96 (0.71 to 1.30)) in the corresponding risk window. Again, similar results were observed in the subpopulation with primary care data but with even wider confidence intervals (table 3, supplement figure S4). Product specific risk estimates did not show any clearly increased risks and were very imprecise (table S7, supplement figure S3). In supplement table S8, we show menstruation disorders in the subpopulation with primary care data after each dose within the risk window at days seven, 28, or 90.

#### Menstruation and bleeding disorders after a positive SARS-CoV-2 test

The risk for the three outcomes was reduced during the first seven days after a positive test (supplement table S9). However, within 90 days, the risk weakly increased, most notably for postmenopausal bleeding (hazard ratio 1.28 (95% confidence interval 0.88 to 1.86)) and premenopausal bleeding (1.45 (0.91 to 2.32)). Of note, the number of cases of premenopausal bleeding was very low. A similar pattern was observed in the subpopulation with primary care data, with slightly higher point estimates for postmenopausal bleeding (supplement table S10).

## Discussion

In this large population-based study of nearly three million women, we observed weak but reasonably precise associations between SARS-CoV-2 vaccination and healthcare contacts for postmenopausal bleeding. Increased risk was observed after the second and third dose in the 8-90 days risk window and was of similar size in the one to seven days risk window after a third dose. This pattern is somewhat unexpected for a causal association. Analyses of associations with individual vaccine products and risk of postmenopausal bleeding provided results that suggest an increased risk with BNT162b2 and mRNA-1273 after the third dose, but suggest a less clear association with ChAdOx1 nCoV-19.

For menstrual disturbance, adjustment for covariates almost completely removed the associations found after vaccination in the crude estimates, and only a weak association remained after the first dose, limited to the one to seven days risk window. Considering the characteristics of this condition, and that the change is measured based on encounters with specialist healthcare in this study, a causal effect limited to this risk window is unlikely.

The number of healthcare contacts for the outcome of premenstrual bleeding were fewer, and risk estimates after vaccination consequently more imprecise. The risk was also notably attenuated by adjustment for covariates and overall did not support an association with SARS-CoV-2 vaccination.

The risk of the three outcomes did not substantially increase after covid-19, although point estimates for postmenopausal bleeding were increased in the 90 day risk window after infection.

### Strengths and weaknesses

The main strengths of our study include the population-based cohort design, large sample size, near complete follow-up, and independent ascertainment of data for SARS-CoV-2 vaccinations and healthcare contacts from nationwide registers with mandatory reporting, in a setting with a universal, tax financed healthcare system. We have adjusted for socioeconomic factors, previous healthcare use, and for several specific medical conditions, including diagnosis of obesity and chronic obstructive pulmonary disease. We have no direct information on ease of access to healthcare, body mass index, or smoking. With a possible exception for postmenopausal bleeding, healthcare contacts for menstrual disorders might have a modest sensitivity. Also, we have no information on whether the healthcare contact was a planned or an acute visit. Time from first symptoms to healthcare contact is probably longer for women with menstrual disturbances and bleeding before menopause than for women after menopausal who have bleeding. Use of the date of healthcare contact for these conditions does not mean that the date of onset of the condition is analysed. The time between onset, start of symptoms, and date of healthcare contact might thus be considerable, making the interpretation of effect of different risk windows challenging. Hence, we might also, especially for menstrual disturbances and premenopausal bleeding, catch some prevalent (before exposure) cases, especially in the one to seven day time window analysed. We are unable to acquire the point in time when a woman enters menopause. Hence, we rely on the physician using the correct codes from the International Statistical Classification of Diseases and Related Health Problems for defining premenopausal bleeding or postmenopausal bleeding. Reverse causation, where women get vaccinated before a planned healthcare contact, is also an issue. Also, women with an ongoing covid-19 infection will probably cancel or postpone planned or semi-acute healthcare contacts.

### Other studies and supportive data

The concern for an association between SARS-CoV-2 vaccination and menstrual or bleeding disturbances in women has been triggered by the large number of spontaneous case reports related to such conditions.^1^ ^2^ ^3^ ^10^ Also, several studies on self-reported menstruation cycles changes after SARS-CoV-2 vaccination have been published.^4^ ^5^ ^6^ ^7^ ^8^ ^9^ ^27^ ^28^ The European Medicines Agency has recommended that heavy menstrual bleeding should be acknowledged as a side effect of both SARS-CoV-2 mRNA vaccines.^12^ However, European Medicines Agency considered that the available data do not support causal association between SARS-CoV-2 mRNA vaccines and absence of menstruation.^12^ The results from the present study are not necessarily contradictive of this labelling, which was mainly based on self-reported survey data and spontaneous case reports. This type of data can be prone to recall bias. Self-reporting might also obtain events that normally would not result in a healthcare contact but might still be sufficiently disturbing to be relevant for the affected women. Self-reporting, as well as health seeking behaviour, can be stimulated by media attention.^10^ ^29^ To the best of our knowledge, no previous large observational study has assessed an association between SARS-CoV-2 vaccination and healthcare contacts for menstrual or bleeding disorders using independent ascertainment of both exposure and outcome.

No clear and specific mechanistic explanation allows for this type of association or supports a general such association with vaccines.^30^ ^31^ An unspecific activation of the immune system might trigger menstruation effects.^11^ Two studies based on self-reported data have reported some associations between human papillomavirus vaccines and menstruation effects.^13^ ^14^ However, a large population-based study found no association between human papillomavirus vaccination and primary ovarian insufficiency.^15^ Menstrual effects are not labelled in any of the influenza vaccines or hepatitis A or B vaccines used in the European Union at present.^13^ ^14^ ^15^

### Conclusions

We observed weak and inconsistent associations between SARS-CoV-2 vaccination and healthcare contacts for postmenopausal bleeding, and even less consistent for menstrual disturbance, and premenstrual bleeding. Extensive adjustment for confounding attenuated most risk estimates. The patterns of association are not consistent with a causal effect. These findings do not provide any substantial support for a causal association between SARS-CoV-2 vaccination and healthcare contacts related to menstrual or bleeding disorders.

**What is already known?**

Large numbers of spontaneous case reports report menstrual disturbance after SARS-CoV-2 vaccination
Studies that used self-reported data indicate menstrual cycle changes after SARS-CoV-2 vaccination

**What this study adds**

No evidence of an increased risk of healthcare contacts for menstrual disturbances or before menopausal bleeding in a cohort of nearly three million women using independent ascertainment of both SARS-CoV-2 vaccination and healthcare contacts
Postmenopausal bleeding and contacts with healthcare had a weak association, but with a pattern that is not expected for a hypothesised underlying causal association between vaccines and postmenopausal bleeding

## Data Availability

Access to similar data requires permission. Apart from ethical approval from the Swedish Ethical Review Authority, researchers will also need approval from each register holder.

## Web extra

Extra material supplied by authors

Web appendix: Online appendix

## Notes

FN, the lead author, affirms that the manuscript is an honest, accurate, and transparent account of the study being reported; that no important aspects of the study have been omitted; and that any discrepancies from the study as originally planned have been explained.

Dissemination to participants and related patient and public communities: We plan to disseminate these research findings to relevant stakeholders by presenting our findings at relevant conferences, by sharing the findings with the European Medicines Agency, the Public Health Agency of Sweden, and other national and international public health and regulatory agencies. Additionally we plan on making press releases to national and international media, as well as making plain language summaries available on the homepages of the Swedish Medical Products Agency and Gothenburg University.

Provenance and peer review: Not commissioned, externally peer reviewed.
