# Supplementary material for: Association between SARS-CoV-2 vaccination and healthcare contacts for menstrual disturbance and bleeding in women before and after menopause: nationwide, register based cohort study
Source: BMJ. 2023 May 3;381:e074778. doi: 10.1136/bmj-2023-074778 (PMC10543813; doi:10.1136/bmj-2023-074778)
Supplement: Supplementary file 1 — Web appendix: Online appendix [file ljur074778.ww.pdf]

SUPPLEMENTAL MATERIALS

Tables and Figures

Supplemental Figures 3

DAG S1. Directed acyclic graph (DAG) of vaccination (exposure) and post-menopausal bleeding from health-care contact (outcome, retrieved from National patient register using ICD-10 diagnosis) 3

DAG S2. Directed acyclic graph (DAG) of vaccination (exposure) and menstrual disturbance and pre-menopausal bleeding from health-care contact (outcome, retrieved from National patient register using ICD-10 diagnosis) 4

Figure S1. Forest plot showing HRs with 95% confidence interval for three menstruation disorders after each dose in the 1-7 and 8-90 days risk windows, among women in a Swedish population cohort. The detailed data are presented in Table 2. 5

Figure S2. Forest plot showing HRs with 95% confidence interval for three menstruation disorders after each dose in the 1-7 and 8-90 days risk windows in the subpopulation with primary care data (Stockholm Region and Västra Götaland Region). The detailed data are presented in Table 3. 6

Figure S3. Forest plot showing HR with 95% confidence intervals for three menstruation disorders after each dose of three vaccine products in the 1-7 and 8-90 days risk windows, among women in a Swedish population cohort. The detailed data are presented in Table 4, S6 and S7. 7

Supplemental Tables 8

Table S1. List of prior comorbidities and prior treatment used as covariates in statistical models 8

Table S2. Distribution of covariates among population with different vaccine status 9

Table S3. Trend of yearly diagnoses of menstruation disorders from 2015 to 2022 in the National Patient Register 12

Table S4. Hazard ratios (HR) for post-menopausal bleeding after each dose in 1-7 days and 8-90 days risk windows in the women without prior hormone treatment therapy in the whole study population and in the subpopulation with primary care data (Stockholm Region and Västra Götaland Region, approximately 40% of total population), among women in a Swedish population cohort. 13

Table S5. Hazard ratios (HR) for post-menopausal bleeding after each dose in 1-7 days and 8-90 days risk windows in the women without prior condition on coagulation diseases in the whole study population and in the subpopulation with primary care data (Stockholm Region and Västra Götaland Region, approximately 40% of total population), among women in a Swedish population cohort. 14

Table S6. Hazard ratios (HR) for menstrual disturbance after each dose in 1-7 days and 8-90 days risk windows, stratified by vaccine product, among women in a Swedish population cohort. 15

Table S7. Hazard ratios (HR) for pre-menopausal bleeding after each dose in 1-7 days and 8-90 days risk windows, stratified by vaccine product, among women in a Swedish population cohort. 17

Table S8. Hazard ratio (HR) with 95% confidence interval (CI) for menstruation disorders after each dose in 7-days, 28-days and 90-days risk windows, in the subpopulation with primary care data (Stockholm Region and Västra Götaland Region, approximately 40% of total population) among women in a Swedish population cohort 19

Table S9. Hazard ratios (HR) with 95% confidence interval (CI) for menstruation disorders in 7-days, 28-days and 90-days risk windows after a positive test for Covid-19, among women in a Swedish population cohort. 21

Table S10. Hazard ratios (HR) with 95% confidence interval (CI) for menstruation disorders in 7-days, 28-days and 90-days risk windows after a positive test for Covid-19 in the subpopulation with primary care data (Stockholm Region and Västra Götaland Region, approximately 40% of total population), among women in a Swedish population cohort. 22

Supplemental Figures

DAG S1. Directed acyclic graph (DAG) of vaccination (exposure) and post-menopausal bleeding from health-care contact (outcome, retrieved from National patient register using ICD-10 diagnosis)

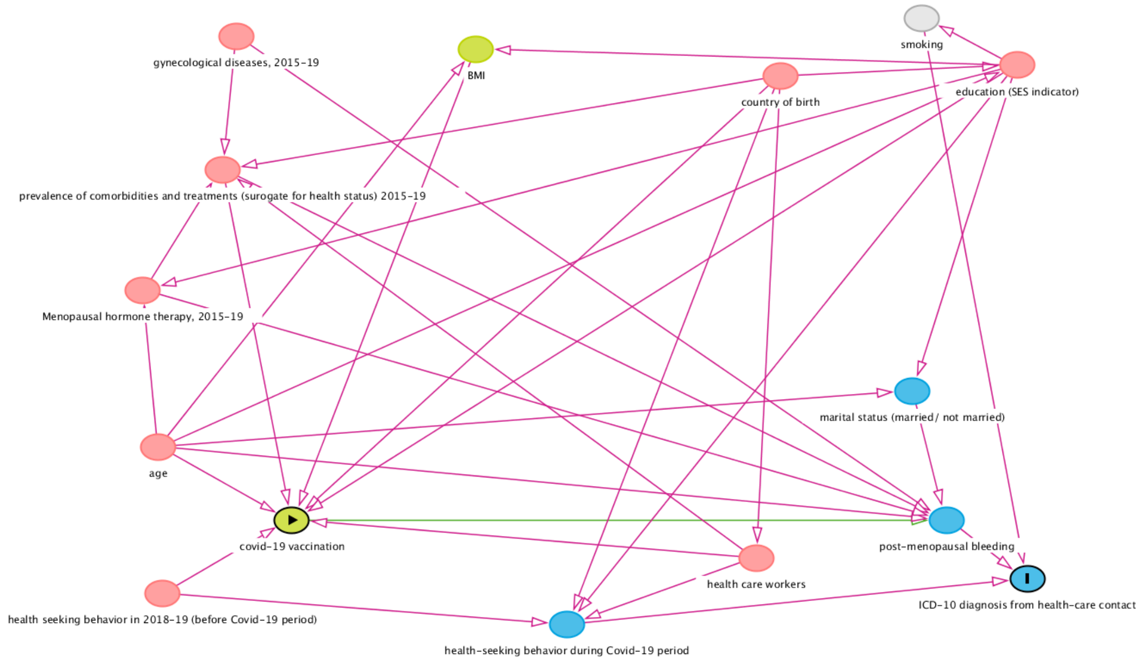

DAG S2. Directed acyclic graph (DAG) of vaccination (exposure) and menstrual disturbance and pre-menopausal bleeding from health-care contact (outcome, retrieved from National patient register using ICD-10 diagnosis)

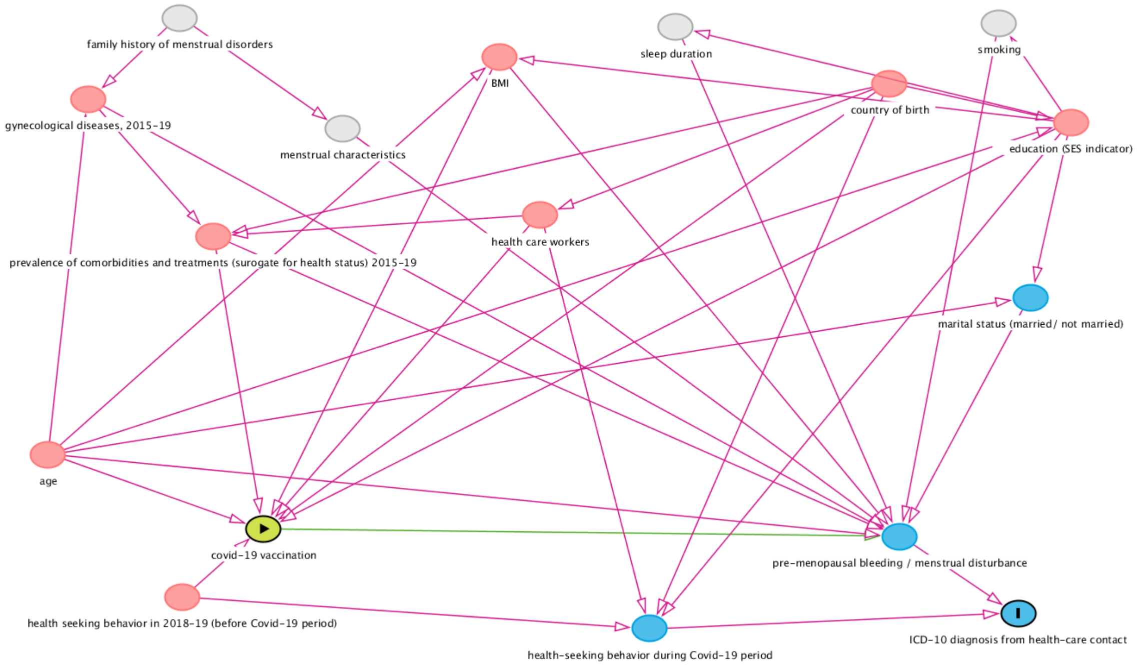

Figure S1. Forest plot showing HRs with 95% confidence interval for three menstruation disorders after each dose in the 1-7 and 8-90 days risk windows, among women in a Swedish population cohort. The detailed data are presented in Table 2.

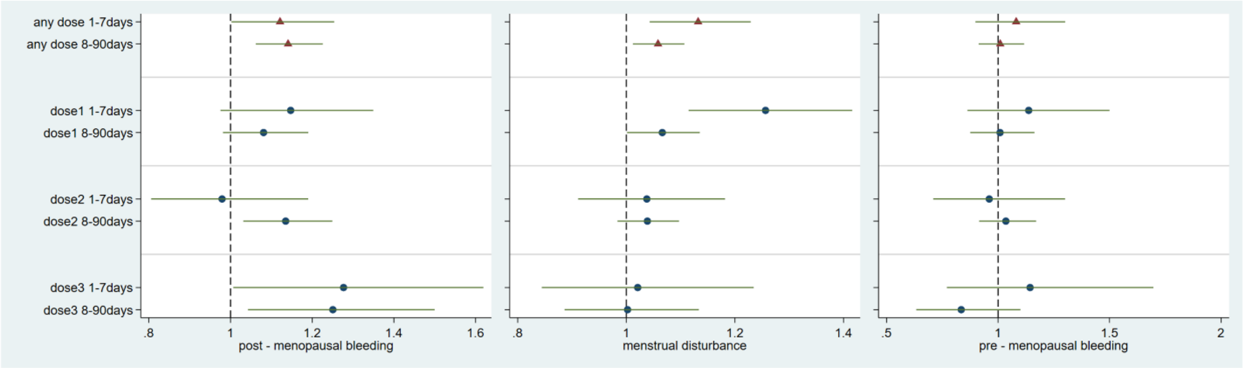

Figure S2. Forest plot showing HRs with 95% confidence interval for three menstruation disorders after each dose in the 1-7 and 8-90 days risk windows in the subpopulation with primary care data (Stockholm Region and Västra Götaland Region). The detailed data are presented in Table 3.

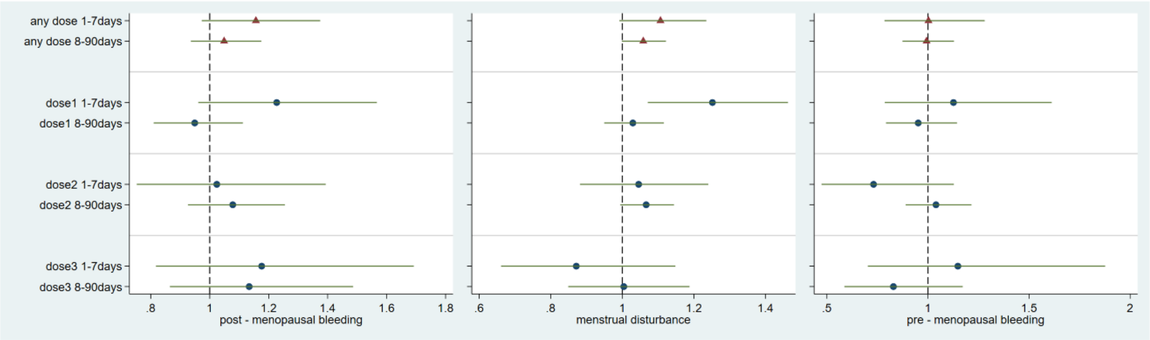

Figure S3. Forest plot showing HR with 95% confidence intervals for three menstruation disorders after each dose of three vaccine products in the 1-7 and 8-90 days risk windows, among women in a Swedish population cohort. The detailed data are presented in Table 4, S6 and S7.

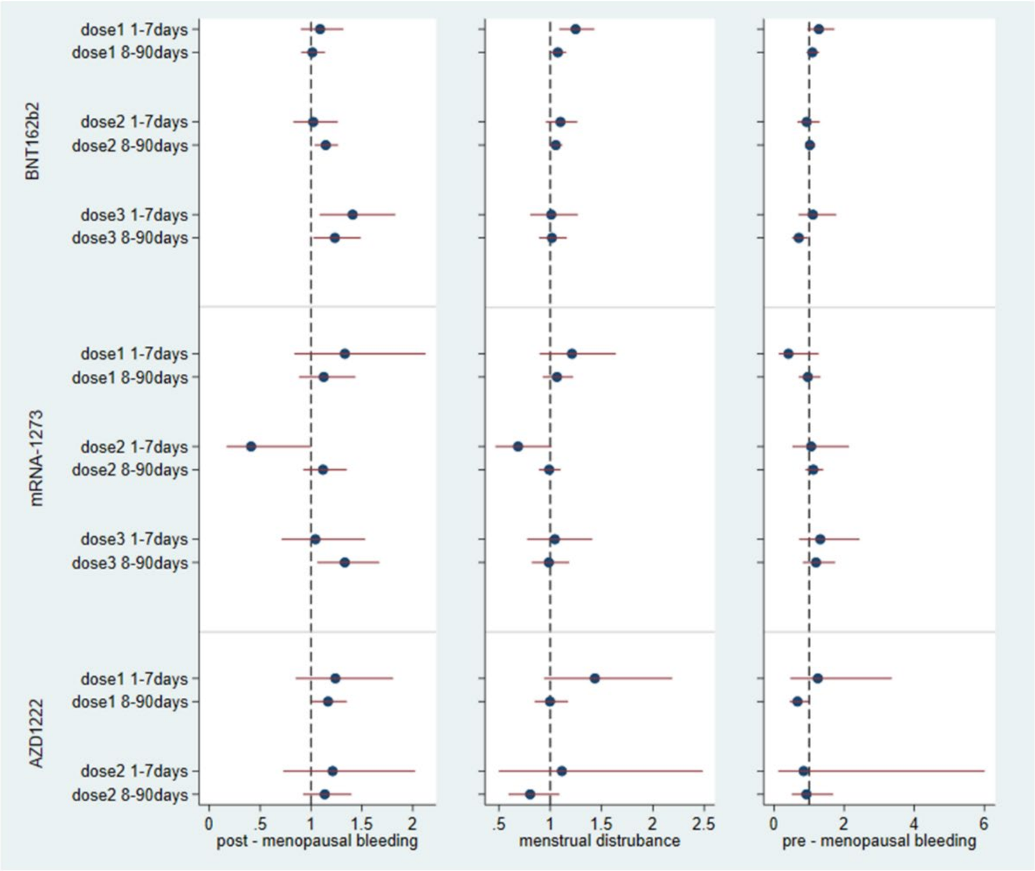

Supplemental Tables

Table S1. List of prior comorbidities and prior treatment used as covariates in statistical models

Prior comorbidities and treatment	ICD-10 from NPR in 2015 to 2019	Prescription medication from NPDR in 2019
Cardiovascular diseases	I05-I09, I20-I51, R001, R011, Q20-Q28	NA
Stroke or transient ischaemic attack	I61 I62 I63 I64 G45	NA
Diabetes (type 1 and 2)	E10, E11	A10A, A10B
Chronic pulmonary disease	J430 J431 J432 J438 J439 J448 J449 J840 J841 J848 J849 J961 J969 E840 E841 E848 E849	NA
Asthma	J45	NA
Chronic kidney disease	N17-N19	NA
Obesity	E66	NA
Autoimmune diseases	M05-M14	NA
Cancer	C00-C97 (except C44)	NA
Coagulation disorders	D65-D69	NA
Polycystic Ovary Syndrome	E28.2	NA
Thyroid disease	E00-E07	H03
Pituitary disorders	E22, E23	NA
Uterine polyps or fibroids	D25, O340	NA
Endometriosis	N80	NA
Pelvic inflammatory disease	N70-77	NA
Antidepressant treatment	NA	N06A
Menopausal hormone therapy	NA	G03C, G03D, G03F
Contraception	NA	G02BA03, G02BB, G03AA, G03AB, G03AC, G03DC02, G03HB01
Tranexamic acid	NA	B02AA02
Oral corticosteroids	NA	H02AB
Anticoagulants	NA	B01AA, B01AB, B01AC, B01AE, B01AF, B01AX
Epilepsy medication	NA	N03A
NSAID/Ibuprofen/salicylates	NA	M01A, N02BA

NA: not applicable.

Table S2. Distribution of covariates among population with different vaccine status

	vaccine status			
Covariates	never vaccinated throughout the study period (N=366 441)	vaccinated with one dose only (N=64 139)	vaccinated with two doses only (N=863 396)	vaccinated with three doses only (N=1 645 367)
age, median (IQR)	31 (17-49)	21 (13-40)	25 (15-43)	53 (41-63)
Employed as a healthcare worker, n (%)				
no	277 644 (75.8)	49 500 (77.2)	633 636 (73.4)	1 052 283 (64.0)
yes	88 797 (24.2)	14 639 (22.8)	229 760 (26.6)	593 084 (36.0)
Country of birth, n (%)				
Sweden	230 156 (62.8)	45 397 (70.8)	675 864 (78.3)	1 425 786 (86.7)
outside Sweden	136 285 (37.2)	18 742 (29.2)	187 532 (21.7)	219 581 (13.3)
Education, n (%)				
primary	61 656 (16.8)	12 012 (18.7)	137 980 (16.0)	203 667 (12.4)
secondary	119 400 (32.6)	16 988 (26.5)	251 327 (29.1)	685 879 (41.7)
tertiary	87 181 (23.8)	11 628 (18.1)	234 332 (27.1)	741 033 (45.0)
unknown	98 204 (26.8)	23 511 (36.7)	239 757 (27.8)	14 788 (0.9)
Cardiovascular disease, n (%)				
no	358 716 (97.9)	62 721 (97.8)	848 071 (98.2)	1 574 821 (95.7)
yes	7 725 (2.1)	1418 (2.2)	15 325 (1.8)	70 546 (4.3)
Stroke or transient ischaemic attack, n (%)				
no	365 005 (99.6)	63 935 (99.7)	861 081 (99.7)	1 630 220 (99.1)
yes	1 436 (0.4)	204 (0.3)	2315 (0.3)	15 147 (0.9)
Diabetes (type 1 and 2), n (%)				
no	358 115 (97.7)	62 784 (97.9)	846 278 (98.0)	1 562 466 (95.0)
yes	8 326 (2.3)	1355 (2.1)	17 118 (2.0)	82 901 (5.0)
Chronic pulmonary disease, n (%)				
no	364 944 (99.6)	63 844 (99.5)	860 950 (99.7)	1 631 930 (99.2)
yes	1497 (0.4)	295 (0.5)	2446 (0.3)	13 437 (0.8)
Asthma, n (%)				
no	358 655 (97.9)	62 165 (96.9)	840 531 (97.4)	1 609 108 (97.8)
yes	7786 (2.1)	1974 (3.1)	22 865 (2.6)	36 259 (2.2)
Chronic kidney disease, n (%)				
no	362 061 (98.8)	63 293 (98.7)	853 675 (98.9)	1 623 789 (98.7)
yes	4380 (1.2)	846 (1.3)	9721 (1.1)	21 578 (1.3)
Cancer, n (%)				
no	363 121 (99.1)	63 595 (99.2)	856 585 (99.2)	1 597 346 (97.1)
yes	3320 (0.9)	544 (0.8)	6811 (0.8)	48 021 (2.9)

Coagulation disorders, n (%)				
no	101 535 (99.1)	19 338 (99.1)	316 515 (99.3)	699 270 (99.2)
yes	931 (0.9)	173 (0.9)	2083 (0.7)	5414 (0.8)
Polycystic Ovary Syndrome, n (%)				
no	100 974 (98.5)	19 265 (98.7)	315 336 (99.0)	700 711 (99.4)
yes	1492 (1.5)	246 (1.3)	3262 (1.0)	3973 (0.6)
Thyroid diseases, n (%)				
no	347 881 (94.9)	61 478 (95.9)	824 562 (95.5)	1 488 662 (90.5)
yes	18 560 (5.1)	2661 (4.1)	38 834 (4.5)	156 705 (9.5)
Pituitary disorders, n (%)				
no	101 647 (99.2)	19 396 (99.4)	316 867 (99.5)	700 755 (99.4)
yes	819 (0.8)	115 (0.6)	1731 (0.5)	3929 (0.6)
Uterine polyps or fibroids, n (%)				
no	99 349 (97.0)	19 104 (97.9)	312 761 (98.2)	684 994 (97.2)
yes	3117 (3.0)	407 (2.1)	5837 (1.8)	19 690 (2.8)
Endometriosis, n (%)				
no	76 850 (75.0)	15 178 (77.8)	255 761 (80.3)	563 494 (80.0)
yes	25 616 (25.0)	4333 (22.2)	62 837 (19.7)	141 190 (20.0)
Pelvic inflammatory diseases, n (%)				
no	89 218 (87.1)	16 980 (87.0)	287 919 (90.4)	655 111 (93.0)
yes	13 248 (12.9)	2531 (13.0)	30 679 (9.6)	49 573 (7.0)
Obesity, n (%)				
no	360 458 (98.4)	62 866 (98.0)	850 257 (98.5)	1617 970 (98.3)
yes	5983 (1.6)	1273 (2.0)	13 139 (1.5)	27 397 (1.7)
Autoimmune diseases, n (%)				
no	361 317 (98.6)	63 270 (98.6)	852 639 (98.8)	1600 333 (97.3)
yes	5124 (1.4)	869 (1.4)	10 757 (1.2)	45 034 (2.7)
Menopausal hormone, n (%)				
no	89 885 (87.7)	17 420 (89.3)	285 302 (89.5)	515 981 (73.2)
yes	12 581 (12.3)	2091 (10.7)	33 296 (10.5)	188 703 (26.8)
Contraception, n (%)				
no	61 549 (60.1)	10 338 (53.0)	136 392 (42.8)	462 269 (65.6)
yes	40 917 (39.9)	9173 (47.0)	182 206 (57.2)	242 415 (34.4)
Anticoagulants, n (%)				
no	358 258 (97.8)	62 837 (98.0)	847 956 (98.2)	1539 216 (93.5)
yes	8183 (2.2)	1302 (2.0)	15 440 (1.8)	106 151 (6.5)
Antidepressant treatment, n (%)				
no	333 938 (91.1)	57 084 (89.0)	767 874 (88.9)	1392 243 (84.6)
yes	32 503 (8.9)	7055 (11.0)	95 522 (11.1)	253 124 (15.4)
Tranexamic acid, n (%)				
no	101 238 (98.8)	19 235 (98.6)	314 856 (98.8)	698 468 (99.1)

yes	1228 (1.2)	276 (1.4)	3742 (1.2)	6216 (0.9)
Oral corticosteroids, n (%)				
no	355 061 (96.9)	61 946 (96.6)	836 386 (96.9)	1551 959 (94.3)
yes	11 380 (3.1)	2193 (3.4)	27 010 (3.1)	93 408 (5.7)
Epilepsy medication, n (%)				
no	94 329 (92.1)	17 878 (91.6)	299 856 (94.1)	648 679 (92.1)
yes	8137 (7.9)	1633 (8.4)	18 742 (5.9)	56 005 (7.9)
NSAID, n (%)				
no	71 677 (70.0)	13 881 (71.1)	240 354 (75.4)	472 989 (67.1)
yes	30 789 (30.0)	5630 (28.9)	78 244 (24.6)	231 695 (32.9)
numbers of specialist outpatient visits, median (IQR)	0 (0-2)	1 (0-3)	1 (0-2)	1 (0-3)
days of inpatient stay, median (IQR)	0 (0-0)	0 (0-0)	0 (0-0)	0 (0-0)
number of primary care visits, median (IQR)	2 (0-8)	2 (0-8)	3 (1-10)	5 (1-14)

Table S3. Trend of yearly diagnoses of menstruation disorders from 2015 to 2022 in the National Patient Register

year	Post-menopausal bleeding		
	Women with diagnosis	Total women aged 45-79 yrs	proportion of diagnosis
2015	11 967	351 767	3.40
2016	11 808	350 206	3.37
2017	12 380	349 602	3.54
2018	12 941	349 415	3.70
2019	13 758	350 210	3.93
2020	13 486	354 500	3.80
2021	15 146	353 832	4.28
2022	14 775	348 650	4.24
	Menstrual disturbance		
2015	41 266	2 423 403	1.70
2016	41 809	2 441 920	1.71
2017	43 422	2 459 150	1.77
2018	43 414	2 474 570	1.75
2019	43 926	2 488 220	1.77
2020	41 281	2 494 993	1.65
2021	43 967	2 500 653	1.76
2022	41 600	2 501 899	1.66
	Pre-menopausal bleeding		
2015	8 171	2 423 403	0.34
2016	8 211	2 441 920	0.34
2017	8 504	2 459 150	0.35
2018	7 793	2 474 570	0.31
2019	8 082	2 488 220	0.32
2020	8 259	2 494 993	0.33
2021	8 686	2 500 653	0.35
2022	8 336	2 501 899	0.33

Table S4. Hazard ratios (HR) for post-menopausal bleeding after each dose in 1-7 days and 8-90 days risk windows in the women without prior hormone treatment therapy in the whole study population and in the subpopulation with primary care data (Stockholm Region and Västra Götaland Region, approximately 40% of total population), among women in a Swedish population cohort.

In the whole study population						
					Crude model [a]	Full model [b]
Vaccine status	Risk windows	Person-years	Cases	Incidence (per 100 000 person-years)	HR (95%CI)	HR (95%CI)
unvaccinated		571 731	2 252	393.9	ref	ref
dose 1	1-7 days	23 712	112	472.3	1.16 (0.95, 1.41)	1.13 (0.93, 1.38)
	8-90 days	137 171	592	431.6	1.12 (1, 1.25)	1.10 (0.98, 1.23)
dose 2	1-7 days	23 453	82	349.6	1.00 (0.79, 1.26)	0.96 (0.76, 1.22)
	8-90 days	275 943	1 160	420.4	1.25 (1.13, 1.4)	1.22 (1.1, 1.37)
dose 3	1-7 days	19 554	93	475.6	1.51 (1.15, 1.99)	1.48 (1.12, 1.94)
	8-90 days	156 437	675	431.5	1.39 (1.13, 1.72)	1.40 (1.13, 1.72)
Subpopulation (Stockholm Region and Västra Götaland Region)						
unvaccinated		222 225	900	405.0	ref	ref
dose 1	1-7 days	8 776	47	535.6	1.30 (0.96, 1.77)	1.29 (0.95, 1.76)
	8-90 days	51 526	206	399.8	1.07 (0.89, 1.29)	1.05 (0.86, 1.27)
dose 2	1-7 days	8 646	28	323.8	1.00 (0.67, 1.49)	0.96 (0.64, 1.44)
	8-90 days	101 601	427	420.3	1.34 (1.12, 1.59)	1.30 (1.09, 1.56)
dose 3	1-7 days	6 952	34	489.0	1.41 (0.91, 2.17)	1.40 (0.90, 2.16)
	8-90 days	5 3382	243	455.2	1.35 (0.98, 1.85)	1.37 (0.99, 1.89)

a. Crude model included no covariates.
b. Full model included age, country of birth, employed as a healthcare worker, marital status, education, health seeking behaviours during 2018-2019 (i.e., number of primary care visits, number of specialist outpatient visits, and days of inpatient stay), and prior comorbidities and treatments listed in table S1.

Table S5. Hazard ratios (HR) for post-menopausal bleeding after each dose in 1-7 days and 8-90 days risk windows in the women without prior condition on coagulation diseases in the whole study population and in the subpopulation with primary care data (Stockholm Region and Västra Götaland Region, approximately 40% of total population), among women in a Swedish population cohort.

In the whole study population						
					Crude model [a]	Full model [b]
Vaccine status	Risk windows	Person-years	Cases	Incidence (per 100 000 person-years)	HR (95%CI)	HR (95%CI)
unvaccinated		645 794	3 134	485.3	ref	ref
dose 1	1-7 days	27 458	166	604.6	1.20 (1.02, 1.41)	1.15 (0.98, 1.35)
	8-90 days	158 839	842	530.1	1.14 (1.04, 1.25)	1.08 (0.98, 1.19)
dose 2	1-7 days	27 168	121	445.4	1.06 (0.87, 1.28)	0.98 (0.8, 1.19)
	8-90 days	319 740	1 557	487.0	1.22 (1.11, 1.34)	1.14 (1.03, 1.25)
dose 3	1-7 days	22 865	128	559.8	1.45 (1.14, 1.84)	1.28 (1.01, 1.63)
	8-90 days	185 870	992	533.7	1.39 (1.17, 1.67)	1.25 (1.05, 1.5)
Subpopulation (Stockholm Region and Västra Götaland Region)						
unvaccinated		252 709	1 341	530.7	ref	ref
dose 1	1-7 days	10 265	75	730.6	1.30 (1.02, 1.66)	1.23 (0.97, 1.57)
	8-90 days	60 177	310	515.1	1.04 (0.89, 1.21)	0.95 (0.81, 1.12)
dose 2	1-7 days	10 120	49	484.2	1.13 (0.83, 1.54)	1.01 (0.74, 1.38)
	8-90 days	118 965	597	501.8	1.21 (1.04, 1.4)	1.09 (0.93, 1.26)
dose 3	1-7 days	8 236	50	607.1	1.41 (0.98, 2.02)	1.18 (0.82, 1.7)
	8-90 days	64 415	378	586.8	1.34 (1.03, 1.74)	1.14 (0.87, 1.49)

a. Crude model included no covariates.
b. Full model included age, country of birth, employed as a healthcare worker, marital status, education, health seeking behaviours during 2018-2019 (i.e., number of primary care visits, number of specialist outpatient visits, and days of inpatient stay), and prior comorbidities and treatments listed in table S1.

Table S6. Hazard ratios (HR) for menstrual disturbance after each dose in 1-7 days and 8-90 days risk windows, stratified by vaccine product, among women in a Swedish population cohort.

	BNT162b2 (Pfizer)					
					Crude model [a]	Full model [b]
Vaccine status	Risk windows	Person-years	Cases	Incidence (per 100 000 person-years)	HR (95%CI)	HR (95%CI)
unvaccinated		1 066 457	9 615	901.6	ref	ref
dose 1	1-7 days	20 889	223	1 067.6	1.46 (1.27, 1.67)	1.25 (1.09, 1.43)
	8-90 days	111 773	987	883.0	1.27 (1.18, 1.37)	1.07 (1.00, 1.16)
dose 2	1-7 days	20 927	218	1 041.7	1.28 (1.11, 1.46)	1.10 (0.96, 1.26)
	8-90 days	234 604	2 497	1 064.3	1.34 (1.27, 1.42)	1.05 (1.00, 1.12)
dose 3	1-7 days	8 160	87	1 066.2	1.22 (0.97, 1.54)	1.01 (0.81, 1.27)
	8-90 days	37 995	440	1 158.1	1.39 (1.22, 1.59)	1.02 (0.89, 1.16)
	mRNA-1273 (Moderna)					
unvaccinated		1 066 457	9 615	901.6	ref	ref
dose 1	1-7 days	4 008	43	1 072.9	1.51 (1.12, 2.05)	1.21 (0.90, 1.64)
	8-90 days	23 209	222	956.5	1.32 (1.15, 1.51)	1.07 (0.93, 1.22)
dose 2	1-7 days	3 639	26	714.5	0.88 (0.59, 1.29)	0.69 (0.47, 1.01)
	8-90 days	42 562	441	1 036.1	1.30 (1.17, 1.44)	0.99 (0.89, 1.10)
dose 3	1-7 days	3 365	50	1 486.1	1.59 (1.18, 2.14)	1.05 (0.78, 1.41)
	8-90 days	13 247	185	1 396.5	1.54 (1.28, 1.85)	0.99 (0.82, 1.18)
	AZD1222 (AstraZeneca)					
unvaccinated		1 066 457	9 615	901.6	ref	ref
dose 1	1-7 days	1 147	22	1 918.1	1.84 (1.20, 2.80)	1.44 (0.94, 2.19)
	8-90 days	12 351	155	1 255.0	1.29 (1.10, 1.52)	1.00 (0.85, 1.17)
dose 2	1-7 days	437	6	1 371.5	1.50 (0.67, 3.34)	1.11 (0.50, 2.48)
	8-90 days	5 179	43	830.2	1.21 (0.89, 1.63)	0.80 (0.59, 1.09)

a. Crude model included no covariates.

b. Full model included age, country of birth, employed as a healthcare worker, marital status, education, health seeking behaviours during 2018-2019 (i.e., number of primary care visits, number of specialist outpatient visits, and days of inpatient stay), and prior comorbidities and treatments listed in table S1.

Table S7. Hazard ratios (HR) for pre-menopausal bleeding after each dose in 1-7 days and 8-90 days risk windows, stratified by vaccine product, among women in a Swedish population cohort.

	BNT162b2 (Pfizer)					
					Crude model [a]	Full model [b]
Vaccine status	Risk windows	Person-years	Cases	Incidence (per 100 000 person-years)	HR (95%CI)	HR (95%CI)
unvaccinated		1 069 353	1 865	174.4	Ref	Ref
dose 1	1-7 days	20 978	47	224.0	1.55 (1.15, 2.08)	1.28 (0.95, 1.72)
	8-90 days	112 328	207	184.3	1.39 (1.18, 1.63)	1.09 (0.93, 1.28)
dose 2	1-7 days	21 033	37	175.9	1.18 (0.84, 1.64)	0.93 (0.66, 1.30)
	8-90 days	236 034	493	208.9	1.42 (1.25, 1.61)	1.02 (0.90, 1.16)
dose 3	1-7 days	8 231	22	267.3	1.52 (0.95, 2.44)	1.11 (0.69, 1.77)
	8-90 days	38 383	71	185.0	1.08 (0.79, 1.48)	0.7 (0.51, 0.96)
	mRNA-1273 (Moderna)					
unvaccinated		1 069 353	1 865	174.4	Ref	Ref
dose 1	1-7 days	4 026	3	74.5	0.55 (0.18, 1.71)	0.41 (0.13, 1.27)
	8-90 days	23 328	42	180.0	1.33 (0.97, 1.83)	0.96 (0.70, 1.32)
dose 2	1-7 days	3 658	8	218.7	1.55 (0.77, 3.13)	1.06 (0.52, 2.13)
	8-90 days	42 827	104	242.8	1.68 (1.34, 2.10)	1.12 (0.89, 1.40)
dose 3	1-7 days	3 407	12	352.2	2.18 (1.18, 4.03)	1.32 (0.71, 2.43)
	8-90 days	13 417	50	372.7	2.00 (1.38, 2.90)	1.19 (0.82, 1.74)
	AZD1222 (AstraZeneca)					
unvaccinated		1 069 353	1 865	174.4	Ref	Ref
dose 1	1-7 days	1 149	4	348.2	1.75 (0.65, 4.71)	1.25 (0.46, 3.35)
	8-90 days	12 386	24	193.8	0.95 (0.63, 1.43)	0.67 (0.44, 1.01)
dose 2	1-7 days	439	1	227.6	1.25 (0.18, 8.92)	0.84 (0.12, 6.01)
	8-90 days	5 205	11	211.3	1.59 (0.87, 2.90)	0.920.51, 1.69)

a. Crude model included no covariates.

b. Full model included age, country of birth, employed as a healthcare worker, marital status, education, health seeking behaviours during 2018-2019 (i.e., number of primary care visits, number of specialist outpatient visits, and days of inpatient stay), and prior comorbidities and treatments listed in table S1.

Table S8. Hazard ratio (HR) with 95% confidence interval (CI) for menstruation disorders after each dose in 7-days, 28-days and 90-days risk windows, in the subpopulation with primary care data (Stockholm Region and Västra Götaland Region, approximately 40% of total population) among women in a Swedish population cohort

Postmenopausal bleeding (45-79 yrs, n=590,271)						
					Crude model [a]	Full model [b]
Vaccine status	Risk windows	Person-years	Cases	incidence rate (per 100 000 person years)	HR (95%CI)	HR (95%CI)
unvaccinated		252 977	1345	531.67	Ref	Ref
dose 1	7 days	10 281	75	729.52	1.28 (1, 1.63)	1.17 (0.91, 1.49)
	28 days	407 940	231	56.63	1.06 (0.91, 1.25)	0.95 (0.8, 1.12)
	90 days	70 637	386	546.46	1.09 (0.95, 1.25)	1.00 (0.87, 1.16)
dose 2	7 days	10 134	50	493.39	1.19 (0.86, 1.63)	1.00 (0.72, 1.38)
	28 days	40 520	183	451.63	1.12 (0.91, 1.37)	0.97 (0.79, 1.2)
	90 days	129 227	647	500.67	1.2 (1.04, 1.38)	1.07 (0.92, 1.24)
dose 3	7 days	8 209	50	609.11	1.47 (1.01, 2.13)	1.2 (0.82, 1.75)
	28 days	32 033	183	571.29	1.36 (1.03, 1.79)	1.14 (0.86, 1.52)
	90 days	72 827	429	589.07	1.35 (1.04, 1.75)	1.14 (0.88, 1.49)
menstrual disturbance (12-49 yrs, n=664,201)						
unvaccinated		446 270	6092	1365.09	Ref	Ref
dose 1	7 days	10 331	169	1635.90	1.41 (1.2, 1.64)	1.24 (1.06, 1.45)
	28 days	41 068	571	1390.39	1.23 (1.12, 1.35)	1.08 (0.99, 1.19)
	90 days	72 005	982	1363.79	1.2 (1.11, 1.29)	1.06 (0.99, 1.15)
dose 2	7 days	9 776	144	1473.03	1.18 (0.99, 1.4)	1.04 (0.87, 1.23)
	28 days	38 877	570	1466.14	1.18 (1.07, 1.3)	1.03 (0.93, 1.13)
	90 days	119 370	1845	1545.61	1.25 (1.17, 1.34)	1.06 (0.99, 1.14)
dose 3	7 days	4 153	61	1468.73	1 (0.76, 1.32)	0.86 (0.65, 1.13)
	28 days	14 324	227	1584.71	1.14 (0.95, 1.36)	0.95 (0.8, 1.13)

	90 days	21 313	349	1637.52	1.22 (1.04, 1.43)	0.98 (0.83, 1.14)
Pre-menopausal bleeding (12-49 yrs, n=664,201)						
unvaccinated		449 008	1210	269.48	Ref	Ref
dose 1	7 days	10 398	32	307.76	1.4 (0.98, 2.01)	1.14 (0.79, 1.63)
	28 days	41 342	113	273.33	1.3 (1.06, 1.59)	1.04 (0.84, 1.28)
	90 days	72 553	194	267.39	1.24 (1.05, 1.46)	0.98 (0.82, 1.16)
dose 2	7 days	9 852	22	223.31	0.98 (0.63, 1.51)	0.74 (0.47, 1.14)
	28 days	39 151	106	270.74	1.16 (0.93, 1.45)	0.87 (0.69, 1.09)
	90 days	120 465	393	326.23	1.39 (1.2, 1.61)	1.01 (0.87, 1.18)
dose 3	7 days	4 209	21	498.92	1.62 (0.98, 2.66)	1.15 (0.7, 1.89)
	28 days	14 523	64	440.69	1.41 (0.99, 2.01)	0.98 (0.69, 1.39)
	90 days	21 623	91	420.84	1.35 (0.97, 1.86)	0.89 (0.65, 1.23)

a. Crude model included no covariates.
b. Full model included age, country of birth, employed as a healthcare worker, marital status, education, health seeking behaviours during 2018-2019 (i.e., number of primary care visits, number of specialist outpatient visits, and days of inpatient stay), and prior comorbidities and treatments listed in table S1.

Table S9. Hazard ratios (HR) with 95% confidence interval (CI) for menstruation disorders in 7-days, 28-days and 90-days risk windows after a positive test for Covid-19, among women in a Swedish population cohort.

				Crude model [a]	Full model [b]
Covid-19 test	Person-years	Cases	Incidence (per 100,000 person-years)	HR (95%CI)	HR (95%CI)
Post-menopausal bleeding (45-74 yrs)					
No positive test	622 812	2 916	468.2	Ref	Ref
7 days after positive test	940	2	212.8	0.46 (0.11, 1.84)	0.44 (0.11, 1.75)
28 days after positive test	2 975	17	571.4	1.25 (0.77, 2.02)	1.18 (0.73, 1.91)
90 days after positive test	4 579	28	611.4	1.36 (0.93, 1.98)	1.28 (0.88, 1.86)
Menstrual disturbance (12-49 yrs)					
No positive test	647 338	6 467	999.0	Ref	Ref
7 days after positive test	1 197	3	250.7	0.26 (0.08, 0.80)	0.24 (0.08, 0.74)
28 days after positive test	3 859	39	1010.7	1.06 (0.77, 1.45)	0.98 (0.72, 1.35)
90 days after positive test	6 136	72	1 173.4	1.26 (1.00, 1.60)	1.18 (0.93, 1.49)
Pre-menopausal bleeding (12-49 yrs)					
No positive test	648 385	1 193	184.0	Ref	Ref
7 days after positive test	1 200	2	166.6	0.90 (0.22, 3.59)	0.81 (0.20, 3.24)
28 days after positive test	3 870	9	232.6	1.27 (0.66, 2.46)	1.14 (0.59, 2.21)
90 days after positive test	6 154	18	292.5	1.63 (1.02, 2.60)	1.45 (0.91, 2.32)

a. Crude model included no covariates.
b. Full model included age, country of birth, employed as a healthcare worker, marital status, education, health seeking behaviours during 2018-2019 (i.e., number of primary care visits, number of specialist outpatient visits, and days of inpatient stay), and prior comorbidities and treatments listed in table S1.

Table S10. Hazard ratios (HR) with 95% confidence interval (CI) for menstruation disorders in 7-days, 28-days and 90-days risk windows after a positive test for Covid-19 in the subpopulation with primary care data (Stockholm Region and Västra Götaland Region, approximately 40% of total population), among women in a Swedish population cohort.

				Crude model [a]	Full model [b]
Covid-19 test	Person-years	Cases	Incidence (per 100,000 person-years)	HR (95%CI)	HR (95%CI)
Post-menopausal bleeding (45-74 yrs)					
No positive test	235 024	1 155	491.4	Ref	Ref
7 days after positive test	393	2	508.6	1.04 (0.26, 4.16)	0.98 (0.24, 3.93)
28 days after positive test	1 265	10	790.2	1.64 (0.88, 3.06)	1.53 (0.82, 2.87)
90 days after positive test	1 966	16	813.9	1.73 (1.05, 2.85)	1.61 (0.98, 2.66)
Menstrual disturbance (12-49 yrs)					
No positive test	263 052	3 857	1466.2	Ref	Ref
7 days after positive test	508	3	591.0	0.41 (0.13, 1.29)	0.39 (0.13, 1.20)
28 days after positive test	1 671	25	1495.8	1.08 (0.63, 1.60)	1.01 (0.68, 1.50)
90 days after positive test	2 700	39	1444.6	1.07 (0.78, 1.47)	1.01 (0.74, 1.39)
Pre-menopausal bleeding (12-49 yrs)					
No positive test	263 682	712	270.0	Ref	Ref
7 days after positive test	510	2	392.2	1.47 (0.37, 5.90)	1.30 (0.32, 5.23)
28 days after positive test	1 679	4	238.2	0.91 (0.34, 2.44)	0.80 (0.30, 2.16)
90 days after positive test	2 712	8	295.0	1.17 (0.58, 2.36)	1.03 (0.51, 2.08)

a. Crude model included no covariates.
b. Full model included age, country of birth, employed as a healthcare worker, marital status, education, health seeking behaviours during 2018-2019 (i.e., number of primary care visits, number of specialist outpatient visits, and days of inpatient stay), and prior comorbidities and treatments listed in table S1.
